# Abrupt response of chemical weathering to Late Quaternary hydroclimate changes in northeast Africa

**DOI:** 10.1038/srep44231

**Published:** 2017-03-14

**Authors:** Luc Bastian, Marie Revel, Germain Bayon, Aurélie Dufour, Nathalie Vigier

**Affiliations:** 1 Université Cote d’Azur, CNRS, Observatoire de la Côte d’Azur, IRD, Geoazur, 250 rueAlbert Einstein, Sophia Antipolis 06560 Valbonne, France; 2 Laboratoire Océanographique de Villefranche sur Mer (LOV, OOV), CNRS, UPMC Univ Paris 06, 181 chemin du Lazaret, 06230, Villefranche sur Mer, France; 3 IFREMER, Unité de Recherche Géosciences Marines, 29280 Plouzané, France; 4 Department of Earth Sciences - Royal Museum for Central Africa, Leuvensesteenweg, 13, B-3080 Tervuren, Belgium

## Abstract

Chemical weathering of silicate rocks on continents acts as a major sink for atmospheric carbon dioxide and has played an important role in the evolution of the Earth’s climate. However, the magnitude and the nature of the links between weathering and climate are still under debate. In particular, the timescale over which chemical weathering may respond to climate change is yet to be constrained at the continental scale. Here we reconstruct the relationships between rainfall and chemical weathering in northeast Africa for the last 32,000 years. Using lithium isotopes and other geochemical proxies in the clay-size fraction of a marine sediment core from the Eastern Mediterranean Sea, we show that chemical weathering in the Nile Basin fluctuated in parallel with the monsoon-related climatic evolution of northeast Africa. We also evidence strongly reduced mineral alteration during centennial-scale regional drought episodes. Our findings indicate that silicate weathering may respond as quickly as physical erosion to abrupt hydroclimate reorganization on continents. Consequently, we anticipate that the forthcoming hydrological disturbances predicted for northeast Africa may have a major impact on chemical weathering patterns and soil resources in this region.

Erosion processes on continents include mechanical erosion and chemical weathering, both of which shape the Earth’s surface and contribute to substantial drawdown of atmospheric carbon via export of organic-rich clay fractions and alteration of silicate minerals. However, while the links between climate and physical erosion rates have been well documented from river chemistry data^1^ and sedimentary records^2^, the response of continental chemical weathering to climate change is not well understood and requires further investigation. At the large scale, the relative importance of physical erosion, temperature, rainfall, vegetation and lithology on chemical weathering over both long (>10^6^ yr) and short (≪10^4^ yr) periods of time is still under debate. Most studies that have established links between climate and silicate weathering were based on the analysis of dissolved phases in modern river basins^3^ or on the reconstruction of past ocean chemistry during the Cenozoic^4^ from marine carbonates or deep-sea ferromanganese deposits. To date, only a few studies have investigated past variations in silicate weathering over short timescales and these have yielded contradictory results^5^,^6^,^7^,^8^,^9^. However, this information is important for predicting the evolution of the short-term carbon cycle and its impact on the Earth’s vital resources. Sediments formed in weathering profiles, exported by large rivers and accumulated on margins, can provide invaluable information on the short-term evolution of weathering at the sub-continental scale.

To provide constraints on the links between hydro-climate and weathering, we have reconstructed the Late Quaternary evolution of rock chemical weathering in the Nile basin, using a marine sediment record recovered from the Nile deep-sea fan, off the coast of Egypt (Fig. 1). Tropical Africa is known to have experienced major hydrological changes during the Quaternary period, which dramatically affected fluvial discharge and particle delivery to the surrounding ocean margins^10^,^11^,^12^. The onset of past humid periods was related to increased summer insolation in the Northern Hemisphere associated with northward migration of the Inter Tropical Convergence Zone (ITCZ), which led to enhanced monsoon precipitations in northeast Africa^13^. In the Nile Basin, the past humid periods of the Pleistocene were systematically characterized by substantial export of Fe-rich sediments to the Mediterranean Sea, essentially reflecting enhanced physical erosion and transport processes from the Ethiopian Highlands^14^ (see also Sup. Mat.). In this region, located about 3000 km upstream from the Nile river mouth, the combination of high altitudes (>3000 m) and heavy monsoonal rains created favorable conditions for continental erosion. The sedimentary sequences that were accumulated at the Nile margin can be used to provide high-resolution temporal information on the links between hydro-climate, erosion and weathering processes on continents.

We focused on a well-dated and well-studied sediment sequence (core MS27PT; chrono-stratigraphy based on 24 ^14^C AMS dated samples; see Fig. 1 and Table S3) that provides a continuous record of terrigenous particles exported from the Nile river basin over the last 32 kyr^15^. The core site lies directly under the influence of the Nile freshwater discharge^15^ (Fig. 1). We studied the finest (<2 μm) clay-size detrital fraction of the sediment for several reasons. Clay minerals form in weathering profiles and are more appropriate than coarser size-fractions, enriched in primary minerals, for investigating past silicate chemical weathering in watersheds. Also, granulometric or mineralogical sorting processes during clay transport are minimized. Due to their small size and density, clays are expected to be delivered from source to sink with minimal transport time. This is supported by previous studies of Nile delta sites^15^,^16^,^17^,^18^, which show that the sedimentation rates and major elements concentrations co-vary with climate proxies with no detectable time lag. Nile river discharge is strongly influenced by the monsoonal floods^15^,^16^,^17^,^18^ and the maximal floods are systematically concentrated within a restricted season in the year, thus limiting the size of the alluvial plain. Diagenetic processes including reverse weathering in deltaic waters may also result in clay formation^19^,^20^ in marine environment. However studies of cores from the Nile Delta (e.g. refs 14, 21) all indicate that the main driver of temporal variations in the sedimentation rate is the rate of physical erosion in the Nile Basin. This might be explained by the massive amounts of terrigenous material deposited by the Nile River in the deep sea fan, which reduce the interaction time between water and minerals. In addition, these sediments contain low level in organic matter (0–1.5% in MS27PT sediments^22^), in contrast to e.g. the Amazon sediments, in which reverse weathering has been evidenced^19^.

In order to reconstruct past variations in chemical weathering and discriminate any potential source effects, we used a combination of weathering proxies, including lithium (Li) isotopes and major element abundances, and sediment provenance tracers such as neodymium (Nd) isotopes. Neodymium isotopic ratios remain largely unaffected by weathering and transport processes, and clay-sized sediments faithfully preserve the Nd isotopic composition of their parent rocks^23^,^24^. In contrast, experimental and field studies have demonstrated significant fractionation of Li isotopes during silicate weathering, with preferential incorporation of ^6^Li in neoformed clay minerals^25^. Recent works illustrate the potential of δ^7^Li (δ^7^Li = (^7^Li/^6^Li)_sample_/(^7^Li/^6^Li)_LSVEC_) − 1) × 1000) as a proxy for determining the intensity or the regime of chemical weathering^7^,^26^. Li in river waters and particles is essentially derived from alteration of silicate lithologies^27^,^28^. In igneous provinces, both river-water and sediment δ^7^Li signatures are usually lower in areas with thick soil sequences (i.e. in transport-limited regimes)^7^,^26^. In contrast, mountainous or glaciated regions (weathering-limited regimes^29^) are usually characterized by high δ^7^Li signatures, except for regions underlain by shales^30^. On continents, the key controls of clay δ^7^Li value are the leaching rates of silicate source rocks and the amounts and Li concentrations of neoformed clays. Recent clay data compilation^4^ indicates that the clay-water Li isotope fractionation factor mainly depends on the Li crystalline site, and not on clay chemical composition or clay type.

In core MS27PT sediments, during the last 32 kyrs, clay Li concentrations are high (51 ± 18 ppm) compared to igneous rocks (6 ppm in basalts and 22 ppm in granites on average^25^). Most recent clays (i.e. <5 kyr) display an average concentration of 59 ppm ± 17 ppm, which is close to the Li content of clays sampled in the Blue Nile watershed downstream (45 ppm ± 22 ppm, Fig. S3). This supports a small impact of reverse weathering during mineral alteration/neoformation, in the mixing zone or after sediment deposition. Elevated Li contents in the clay fractions are mainly due to the dominance of smectite (>75%, Fig. S3). Kaolinite and illite can contain similar amounts of Li as smectite but their abundance remains low (<20%) in this core. Clay fractions could contain some Fe oxy-hydroxides, although their greater density would favor their physical separation (see Sup. Mat. Fig. S3). Their contribution to the clay Li budget is expected to be negligible also because, when formed at low-T, they contain low Li levels (≈1 ppm)^31^. The role of Fe- oxyhydroxide phases onto the δ^7^Li is mostly significant in rivers draining areas with limited soil sequences, such as in those encountered at high latitude regions^32^,^33^. During clay crystallization, experimental smectite syntheses show that Li is rapidly “locked” into the octahedral sites during clay crystallization^25^, and that negligible Li adsorption on smectite grains is expected in the marine and subseafloor environments^25^,^34^. Nevetheless, we have developed a chemical procedure that, prior to clay digestion, achieves quantitative removal of any exchangeable Li. Therefore, the Li concentrations and isotope compositions determined during the course of this study correspond to “structural” Li, i.e. Li ions located within clay octahedral sites^25^.

In core MS27PT, clay εNd (εNd = (^143^Nd/^144^Nd)_sample_/^143^Nd/^144^Nd)_sdt_) − 1) × 10000) display significant variations between 32 and 0.5 kyr BP (Table S2, Figs 2 and 3), that follow the curve for 15°N summer insolation intensity, the main forcing mechanism for rainfall in northeast Africa^35^. Downcore εNd variations can be explained by changes in the relative proportions of clays originating from the Ethiopian Traps (εNd ≈ 0), the Equatorial craton (εNd ≈ −30), and the Saharan region (εNd ≈ −10)^36^. During the two humid periods (from ~15 to 7 kyr BP for the African Humid Period, and ~32 to 25 kyr BP), clay εNd are similar to the Ethiopian Traps rock values. When combined with evidence for correspondingly high sedimentation rates, this observation points towards enhanced delivery (>90%) of detrital material issued from the Ethiopian Highlands (Figs 1 and 2). In contrast, the lowest εNd values recorded during arid periods indicate larger proportions of clays derived from the Saharan region and/or the central African cratons. In addition, the clay εNd profile follows the δD_wax_-based precipitation signal reconstructed from Lake Tana^35^, in the northern part of the Ethiopian Traps, which indicates a substantial increase of rainfall in this area during the African Humid Period. Overall, the tight coupling between clay provenance and precipitation signals over the last 30 thousand years indicates a rapid response of physical erosion to increased or decreased monsoon rainfall intensity, thus confirming the high transport efficiency of clays within the Nile river basin.

Three different proxies for chemical weathering intensity (clay δ^7^Li, K/Ti, Mg/Ti) also exhibit climate-related downcore variations (Fig. 2), with lower, and broadly similar, values during the two humid periods, and distinct signatures during the arid ones. Titanium is an immobile element and is incorporated into secondary minerals, including clays. In contrast, potassium is highly mobile during weathering processes, and hence is usually depleted in soils. Mg and Li concentrations are usually positively correlated in clays (Li substitutes to Mg in octahedral sites) and both elements are sensitive to leaching and neoformation processes. In core MS27PT, the low K/Ti and Mg/Ti ratios measured during humid periods may reflect more intense leaching in soils due to enhanced precipitation. Part of the observed variations could, however, also be explained by changes in sediment source, as inferred from the Nd isotope data. For instance, low K/Ti ratios could be inherited, at least in part, from the K-poor geochemical signature of the Ethiopian basalts. However, K/Ti correlates positively with Mg/Ti in core MS27PT. Because basalts are typically enriched in Mg, the co-variation between K/Ti and Mg/Ti observed downcore (Fig. S4) most likely indicates that these variations are controlled mainly by changes in weathering conditions, rather than shifts in sediment sources. The lower K/Ti values recorded during humid periods indicate that the corresponding sediments experienced a more intensive leaching process during their formation. In contrast, during arid periods, high Mg/Ti ratios support the hypothesis that clay neoformation was favored in the Ethiopian Traps, possibly due to a higher residence time of these particles within the watershed, in agreement with the lower physical erosion rates highlighted by other studies^17^.

Clay δ^7^Li also displays small but significant variations between arid and humid periods. During the two humid periods covered by our sediment record, exported clays are characterized by low (1.7‰ on average) and constant (2σ = 0.6‰) δ^7^Li values. In contrast, during the 25–15 kyr arid period, clay δ^7^Li values are higher (2.7‰ on average) and exhibit greater variability (2σ = 1.4‰). Compared to dominant silicate rocks that outcrop in the Nile Basin (see Fig. S4), clays are preferentially enriched in ^6^Li, as expected for secondary phases formed on continents^7^. The particular behavior for Li isotopes during arid versus humid periods is consistent with K/Ti and Mg/Ti variations, reflecting mitigated effects of leaching processes in weathering profiles, which release dissolved Li to the surrounding waters, and clay neoformation, which immobilizes Li (and Mg) in specific soil horizons^37^. To further support this hypothesis, we use a simple model of alteration considering (1) homogenous δ^7^Li at the scale of the source region, and (2) that all of the Li released by dissolution of the source rocks is either incorporated into secondary phases in soils or is transported to river water^38^,^39^ (Fig. S5). Using this model, it can be shown that clay δ^7^Li is an inverse function of F_lea_/F_sp_: the ratio of the Li flux related to bedrock leaching (F_lea_), to the Li flux associated with secondary phase formation (F_sp_). Thus, our model indicates that the low and rather constant value of 1.7‰ during humid periods is best explained by (1) a small isotope fractionation factor during clay formation and (2) high F_lea_/F_sp_ ratios (Fig. S5). Conversely, the low and highly variable clay δ^7^Li values during arid periods are consistent with lower leaching (dissolution) rates and/or higher clay formation rates. Taken altogether, the observed concomitant temporal variations in δ^7^Li, K/Ti and Mg/Ti from arid to humid periods suggest that mineral leaching and/or clay formation rates responded rapidly (without any apparent delay) and significantly to changes in rainfall intensity over the Nile basin. Another possibility could be that, during humid periods, clays come from deeper soil horizons (because high physical denudation have removed the top layers) with lower δ^7^Li values. Previous investigations have shown indeed that δ^7^Li of bulk soil samples can vary as a function of depth^37^,^40^,^41^,^42^,^43^. However, when compiling all the published soil data, no clear δ^7^Li systematics appears between the upper and deeper soil horizons at the scale of river catchments: some soils exhibit constant δ^7^Li as a function of depth while others may display variable compositions with enrichment or depletion in ^7^Li^37^. Despite this uncertainty, both interpretations are consistent with thinner soils during humid periods, and production of new secondary phases during the following arid times.

Interestingly, our three weathering proxies (clay δ^7^Li, K/Ti and Mg/Ti) also appear to exhibit some variability at shorter millennial to centennial timescales, in particular during the so-called Heinrich stadial, which correspond to “cold” periods in the Northern Hemisphere^44^,^45^, and which have been documented as arid events in tropical Africa^21^,^46^ (Fig. 3). In core MS27PT, Heinrich stadial 1, 2 and 3 (HS1, HS2 and HS3), the Last Glacial Maximum (LGM) and the Younger Dryas (YD) are all characterized by higher Ca/Fe ratios of bulk sediments^15^ (reflecting marine/terrigenous ratio at a high resolution) and a significant increase in clay δ^7^Li. As shown in Fig. 3, the timing of these episodes matches the extreme arid events recently documented in marine sediments from Western Africa^46^ and from the Nile delta^21^. The onset of arid conditions in northern Africa during these periods is generally attributed to a weakening of the Atlantic Meridional Overturning Circulation (AMOC) and to a southward ITCZ migration, which led to severely reduced inputs of Atlantic-derived moisture in the Nile basin. During these arid episodes, clay δ^7^Li increase in phase with the export of dust to sites in West Africa^46^. Except for HS3, Nd isotopes do not appear to have varied significantly during these events suggesting that sediment provenance did not change significantly. According to our model, high clay δ^7^Li values during arid events are consistent with lower leaching rates (F_lea_). Similarly, clay Mg/Ti and K/Ti ratios both exhibit an increase during HS1 and HS2, supporting a reduction in the release rate of mobile elements during rock alteration. The two highest clay δ^7^Li values (4.0‰ and 3.6‰, at 22.7 and 21.4 kyrs cal BP respectively) were measured during the LGM. This could be due to extreme aridity associated with extended glaciers in Equatorial East Africa^47^.

Taken together, our proxies indicate that chemical weathering responded abruptly to past climate change in the Nile Basin in the last 32,000 years, most particularly during the short periods that corresponded to punctual arid events in northern Africa. These results thus strongly suggest that past climatic changes over centennial to millennial timescales had an impact on erosion processes, demonstrating a tight coupling between short-term hydroclimatic oscillations, physical erosion and silicate weathering on continents. The chosen sample size (1 cm) in the MS27PT core corresponds to a resolution of ~400 to ~1300 yrs during arid periods, when the δ^7^Li signal is the most variable. This age range gives an approximate order of magnitude for the minimum response time of silicate weathering to climate change in the Nile Basin. This result may seem small compared to the expected residence of soil in a watershed as large as the Nile River Basin. Previous studies have shown that large tropical catchments in Africa (e.g. the Congo Basin) have soils that are in steady-state (i.e. with a constant thickness) over a long residence time (probably >1 Ma)^48^. However, in the Congo Basin, rapid responses of chemical weathering to climate change have been highlighted for the last 20 kyrs^5^, suggesting that young soils are reactive and can imprint the chemical signature of clay-rich sediment carried by the river and deposited offshore. In the Nile Basin, during the period studied, most clays come from the Ethiopian Traps (based on εNd), and it is possible that the soil residence time in this mountainous and volcanic region is short on average, due to high physical erosion rates. The temporal correlation observed between weathering and climate proxies suggests that time lags are negligible, within uncertainties. Thus, the rapid response of alteration in this region suggests that Ethiopian soils are in transient state during arid periods, and/or that the return to steady-state can be reached quickly after a change in climate conditions, such as it has been shown for other basaltic regions impacted by the last glaciation^49^.

The strong reactivity of soils in the Nile basin suggests that the future variations in rainfall intensity predicted for northern Africa^50^ will have a concomitant impact on its soil resources because weathering processes control the recycling and release of key macro- and micro- nutrients to soils and freshwaters. Since continental alteration can be a significant CO_2_ sink over short timescales^51^, our results also suggest that rapid variations in chemical weathering at large scale should be included in the modeling of the impact of future climate change.

## Methods

Each sample of the MS27PT core analyzed in this study corresponds to a 1-cm cut along the width of the core, i.e. about 1 g of bulk sediment. The collected sediment was sieved at 63 μm and then leached for a few minutes using ultra-pure 1N hydrochloric acid in order to remove carbonates, following the method described in ref. 15. The leaching removed less than 4% lithium from the <63 μm fraction, in agreement with experimental results showing that the proportion of exchangeable Li of smectite in marine environments is negligible^34^. The residue was washed three or four times with 50 ml of ultra-pure water (Milli-Q^®^). The clay fraction was then extracted by decantation of the de-carbonated sediments in 50 ml of ultra-pure water with 60 μl of sodium hexametaphosphate. This procedure resulted in a good separation of the clay fraction in samples from both the arid and humid periods, as highlighted by the numerous granulometry measurements that we performed (see example shown in Fig. S1). The clay fraction was finely crushed in an agate mortar and 10 mg of the powder was then completely mineralized using a concentrated HF/HNO_3_/HCl solution. This enabled total dissolution of the silicate and organic phases, as demonstrated by the absence of residue after 20 min of centrifuging at 4000 rpm. For two of the samples, residue was observed, extracted and dissolved with HNO_3_ and/or HF before being mixed with the rest of the solution and centrifuged again. The solution was evaporated and the residue dissolved in HCl 1 N. For major and trace element analyses, an aliquot of the solution was diluted 300 times and analyzed by ICP-AES. For the lithium isotope analyses, the solution was purified beforehand to allow recovery of only the lithium. For this, a solution containing ≈60 ng of Li was introduced on a cationic resin column and eluted with HCl 1N, following the method published in ref. 25. This separation was performed twice. Total cationic concentration for each sample (in meq) was constrained to be less than 10% of the total cationic capacity of the column resin.

Lithium isotope measurements were performed using a Neptune Plus (Thermofisher) multi-collector inductively coupled plasma spectrometer (MC-ICP-MS) at the Ecole Normale Supérieur de Lyon (National Facilities). The Aridus II desolvating system was added before ionization for an increased sensitivity. We used the jet and X cones, as described in ref. 52. To correct for instrumental mass bias, we used the standard bracketing technique with the LSVEC standard. The sensitivity was 1 Volt/ppb, and the solution was diluted to 5 ppb for analysis. The total procedure blank was systematically small (<0.02%), of 10 pg maximum. The accuracy of the isotopic analysis was verified several times during each measurement session using Li7-N, Li6-N pure Li solutions^53^, seawater and rock reference materials. Without separation chemistry, mean δ^7^Li values of 30.2‰ ± 0.1‰ (2σ_n_, n = 18), −7.8‰ ± 0.2‰ (2σ_n_, n = 3) were obtained for Li^7^-N and Li6-N respectively, which compare well with published and theoretical values^53^. After chemical purification, the mean values for δ^7^Li were 30.3‰ ± 0.3‰ (2 SD, n = 13), 31.0‰ ± 0.4‰ (2 SD, n = 5) and 4.6‰ ± 0.2‰ (2 SD, n = 2) for Li7-N, Seawater and BE-N basaltic rock, respectively, which also compare well with published values. One clay sample was analyzed 14 times (14 different powder aliquots) and a second clay sample was analyzed five times with five different chemical separation (same aliquot), yielding values of 5.7‰ ± 0.2‰ (2 SD) and 1.60‰ ± 0.3‰ (2 SD), respectively (Fig. S2 and Table S1).

Neodymium was purified by conventional ion chromatography^5^. Isotopic measurements were performed at the Pôle Spectrométrie Océan (Brest, France) using a Thermo Scientific Neptune multi-collector ICP-MS. Nd isotopic compositions were determined using sample-standard bracketing, by analysing JNdi-1 standard solutions every two samples. Mass bias corrections were made using the exponential law and using ^146^Nd/^144^Nd = 0.7219. Mass-bias corrected values for ^143^Nd/^144^Nd were normalized to a JNdi-1 value of ^143^Nd/^144^Nd = 0.512115^54^. Repeated analyses of bracketed JNdi-1 standard solutions during the course of this study yielded ^143^Nd/^144^Nd of 0.512117 ± 0.000012 (2 SD, n = 16), corresponding to an external reproducibility of ~ ± 0.23ε (2 SD). Epsilon Nd values (ε_Nd_) were calculated using ^143^Nd/^144^Nd = 0.512630^55^.

## Additional Information

**How to cite this article:** Bastian, L. *et al*. Abrupt response of chemical weathering to Late Quaternary hydroclimate changes in northeast Africa. *Sci. Rep.* **7**, 44231; doi: 10.1038/srep44231 (2017).

**Publisher's note:** Springer Nature remains neutral with regard to jurisdictional claims in published maps and institutional affiliations.

## Supplementary Material

Supplementary Information

## Figures and Tables

**Figure 1:**
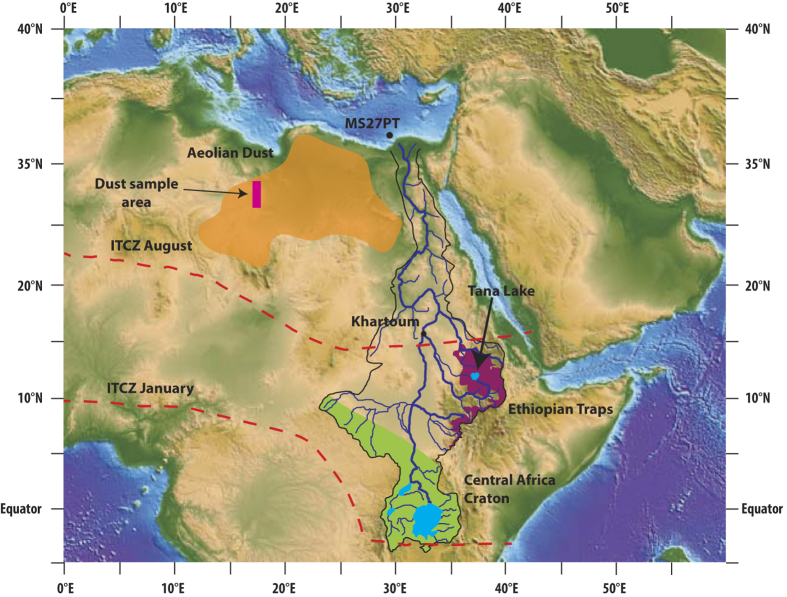
Map of the Nile catchment and location of core MS27PT (N31°47.90′; E29°27.70′, 1389 m water depth) (From Revel *et al*.^15^,^17^,^22^; Copyright given to Editor). The Blue Nile tributary drains the Ethiopian Traps (Highlands), which are mostly composed of basaltic rocks (purple). The Equatorial tributary of the Nile drains the African Craton from Victoria Lake to Khartoum. Rocks are Precambrian metamorphics (in green)^36^. The orange area represents the source region of most aeolian dust^56^,^57^. The rectangle corresponds to the localization of the analyzed dust samples. In summer, solar radiation causes the North African landmass to heat up, inducing a low-pressure zone (ITCZ, red dashed lines) that attracts moist air from Atlantic and Indian Oceans (monsoon).

**Figure 2:**
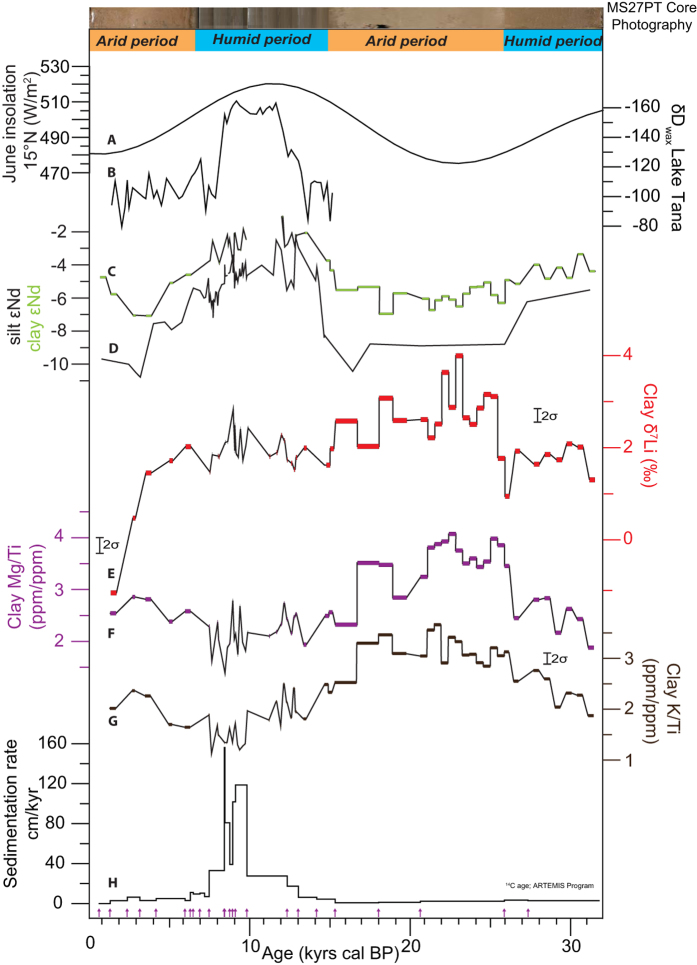
Paleo-variations in (**A**) June insolation at 15°N^58^; (**B**) the hydrogen isotopic composition of leaf wax extracted from a sedimentary core in Lake Tana^58^; (**C**) and (**D**) the Nd isotope compositions of the clay fraction (green, this study) and the silt fractions (black^15^) in MS27PT; (**E**) the Li isotope composition of the MS27PT clay fraction; (**F**) and (**G**) the K/Ti and Mg/Ti ratios of the MS27PT clay fractions, and (**H**) the sedimentation rate in cm/year^15^,^17^,^22^. A photograph of the MS27PT core is shown for comparison, with the humid periods characterized by brown/grey sediments, enriched in Fe-rich silicate minerals, and the arid period characterized by paler sediments due to a dominance of biogenic carbonates (foraminifera).

**Figure 3:**
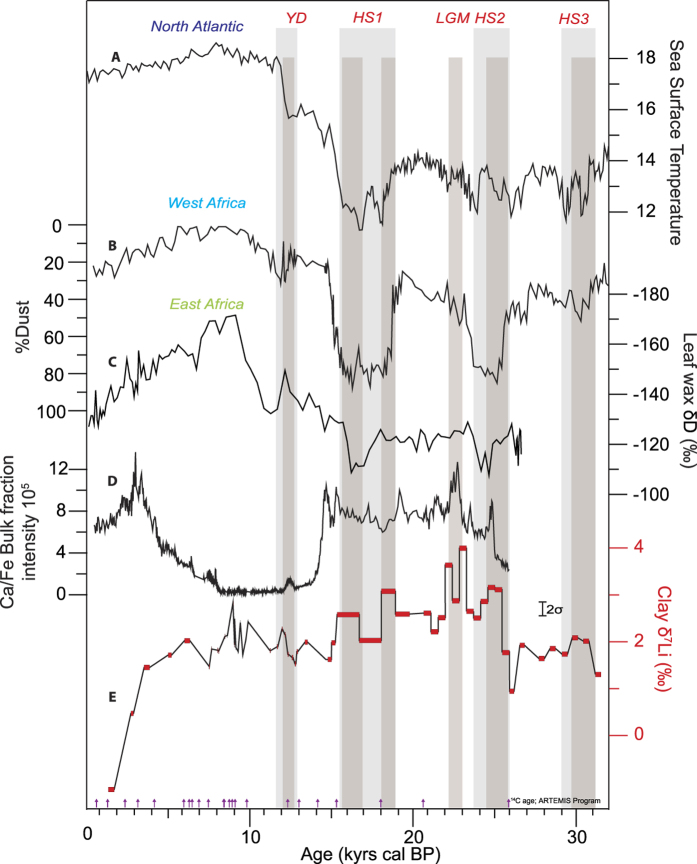
Paleo-variations in (**A**) Sea Surface Temperature (SST, °C) reconstructed for the North Atlantic^59^; (**B**) the estimated percentage of dust in core GeoB9508, close to Gibraltar^46^; (**C**) the hydrogen isotopic composition of leaf wax in core GeoB7702-3, close to Sinaï^21^; (**D**) the Ca/Fe ratio in the MS27PT bulk fractions, measured by XRF core scanner^15^; and (**E**) the Li isotopic composition of the MS27PT clay fractions. HS: Heinrich stadials; LGM: Last Glacial Maximum; YD: Younger Dryas. Light grey bars show YD and HS based on Atlantic records only. Dark grey bars highlight the short characterized by a systematic increase of the clay δ^7^Li value. Since 3 kyrs, clay δ^7^Li decrease is potentially due to anthropic activity in the Nile Basin and is not discussed in this study.
