# Supplementary material for: Abrupt response of chemical weathering to Late Quaternary hydroclimate changes in northeast Africa
Source: Sci Rep. 2017 Mar 14;7:44231. doi: 10.1038/srep44231 (PMC5349522; doi:10.1038/srep44231)
Supplement: Supplementary Information [file srep44231-s1.pdf]

Abrupt response of chemical weathering to Late Quaternary hydroclimate changes in northeast Africa

Luc Bastian[1,2*], Marie Revel[1], Germain Bayon[3,4] , Aurélie Dufour[2], Nathalie Vigier[2]

[1] Geoazur, Université de la Cote d'Azur, CNRS, OCA, IRD, Geoazur, 250 rue Albert Einstein, 06500 Valbonne, France

[2] Laboratoire d'Océanographique de Villefranche sur Mer (LOV, OOV) CNRS, UPMC, 181 chemin du Lazaret, 06320, Villefranche sur Mer, France

[3] IFREMER, Unité de Recherche Géosciences Marines, 29280 Plouzané, France

[4] Department of Earth Sciences - Royal Museum for Central Africa, Leuvensesteenweg, 13, B-3080 Tervuren, Belgium

*corresponding author: bastian@geoazur.unice.fr

SUPPLEMENTARY MATERIAL

Nile basin study area and Sampling

The Nile River is located in north-eastern Africa and is the longest river in the world. It flows over 6700 km, over 31° of latitude, and has a watershed area of 3,000,000 km^2 [1]. The Nile watershed is essentially composed of two main catchments, both of silicate lithology[2]: the Ethiopian highlands, or traps, are mainly composed of basaltic volcanic rocks and are drained by the Blue Nile and the Atbara rivers; the Equatorial craton is mainly composed of gneiss and granulite rock and is drained by the Bahr el Jabel River. This river joins the Sobat River to form the White Nile[3].

At its mouth, the Nile River has a unimodal discharge curve over a year, with summer floods essentially linked to the seasonal monsoonal circulation over the Ethiopian highland sources[2]. Thus, the tropical climate of the Nile Basin is marked by African monsoon precipitation fluctuation. The seasonal change in monsoon precipitation in the Nile Basin is controlled by the convergence of winds from both hemispheres in the InterTropical Convergence Zone (ITCZ). Much of the rainfall over North-East Africa originates in the tropical Atlantic and southern/western Indian Ocean[4,5]. Mean annual precipitation greater than 3.2 mm/day was recorded by the Tropical Rainfall Measuring Mission at Lake Tana for the period 1998-2010[6]. At present, the monsoon occurs during the summer in Northern hemisphere, and the location of ITCZ is linked to the maximal insolation and oscillates north-south during the year. In the past, because long-term changes in the insolation maxima are modulated by changes in the Earth's precessional and obliquity cycles, the north-south migration of the African monsoonal rain belt followed the precessional cycles, with further northward ITCZ penetration during precessional minima[7]. High summer insolation in the early and mid-Holocene enhanced the thermal contrast between land and sea, producing higher rainfall than at present across North Africa, leading to the African Humid Period (AHP) between ~15 and 6 ka[6]. The AHP was evidenced in several sedimentary records[8,9] and mostly concerned the Ethiopian Traps region[10,11].

The MS27PT core was collected in 2004 during the Mediflux MIMES oceanographic cruise, in the Nile deep-sea-fan. The core was collected on along the continental slope at a depth of 1389 meters, from a non-channelized slope located 90km outward from the mouth of the Rosetta Nile branch[10,12]. The ability of this sediment core to monitor past hydrology and weathering changes in the Nile basin has been demonstrated in several sedimentology and geochemistry studies[13,14].

The core site is located adjacent to Rosetta river outflow, resulting in high accumulation rates of terrigenous sediment at the site. Detailed bathymetric and high resolution seismic profiles (eight oceanographic cruises[15]) demonstrated that the Rosetta branch has been active since 115 ka and that this western part of the delta has continuously received sediments from the Nile River[12,13,16]. The sediment loads of each tributary have a distinct Nd isotopic composition reflecting the geology of the catchments. Thus, temporal changes in both Nd signature and sedimentation rate have allowed reconstructing changes in the terrigenous contribution in relation to the north-south oscillation of rain belt[10,12–14].

The influence of the sea-level rise on the nature and the rates of sedimentation has already been investigated in the MS27PT core[10]. The Nile continental shelf is about 30 km wide, with a shelf edge located around 200m water depth (Rouillard, 2010). The post-glacial sea-level rise that started around 20 kyrs with the MeltWater Pulse-1A (MWP-1A started 14,650 years ago and ended before 14,130 years ago, coeval with the Bølling warming[17]. During the LGM, global sea level was lowered by about 120 m, allowing direct connection between the Rosetta fluvial mouth and the Rosetta turbidite system. As sea-level rise proceeded, the distance between the canyon head and the main source of clastic sediments (the Nile estuary mouth) progressively increased, to reach a maximum around 11.5-8 ka, when the shoreline was situated about 5-50 km landward of its present-day position (Stanley & Warne, 1993). As a result, if the distance from the fluvial outlet to the shelf edge was the dominant controlling parameter, one could expect higher sedimentation rates during the LGM along the Rosetta deep-sea fan compared to the last deglacial period and the Holocene. This is not the case. In the same way, [18] have studied more than 105 cores or sections distributed all along the Nile delta for the last 12,000 years. They conclude that Nilotic hydrology and particle flux are the main driver of sedimentation rate variations.

The MS27PT core is characterized by continuous hemipelagic sedimentation over 7m (100 kyr). The present study focuses on the uppermost part of this sediment sequence (433cm), which covers the last 32,000 years but exclude the last 3,000 years, potentially impacted by anthropic activity. The published age models for the period studied are based on 22 AMS^{14}C dates[10,13] (Table S3). The age models as deduced from radiocarbon dating were calibrated to calendar ages using the CALIB 7.0 program[19] and were corrected for a marine reservoir age difference of 400 years for the Holocene, 560 years for the Bølling/Allerød and 800 years during the deglaciation[20].

Heinrich Events

Rapid climate changes are associated with the Dansgaard–Oeschger (D-O) cycles or Heinrich Stadia (HS) and are superimposed on the longer-term orbital climate trends[21,22]. Among them, Heinrich events are recorded at the end of coldest stadial periods of the D-O cycle in the North Atlantic Ocean and were accompanied by massive iceberg formation and extension of the sea ice in North Atlantic[21,22]. Such disturbances of the hydrological conditions of surface waters reduced deep water formation and thus modified Atlantic meridional overturning circulation[23,24]. In tropical Africa, the impact of these events is still under debate but they are expressed as the dustiest periods of the last glacial due to rapid increases in aridity and wind strength in the Sahel[25,26].

Methods

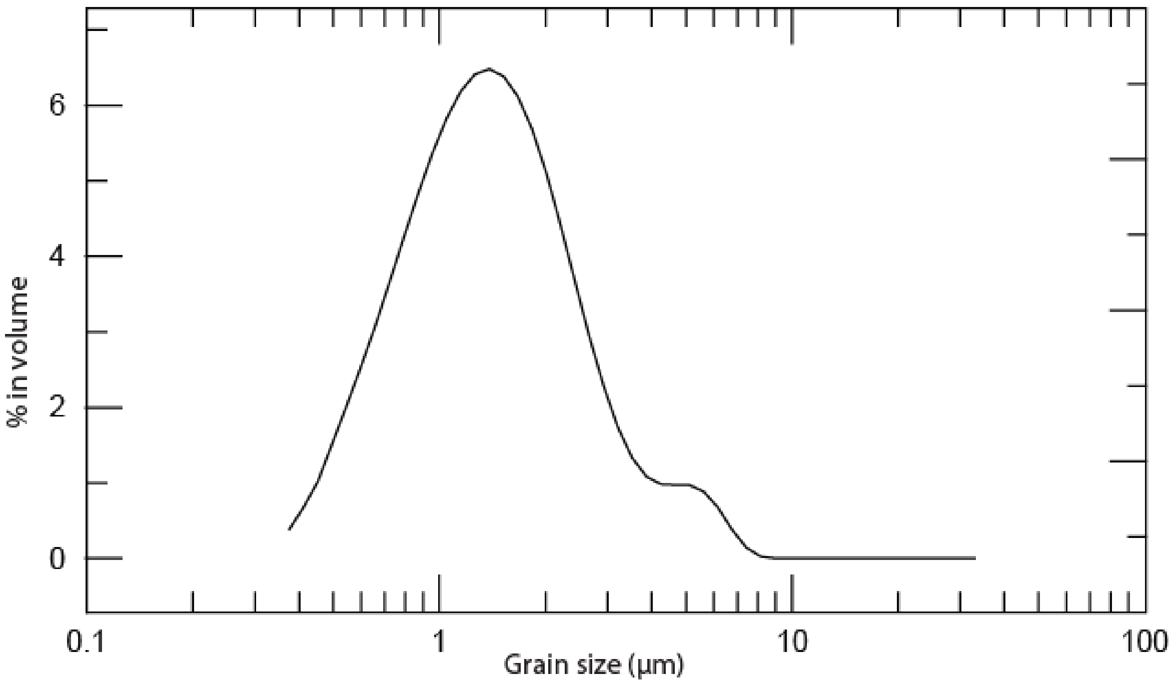

Figure S1: *Proportion of particles (in volume %) as a function of grain size after the decantation procedure for the sample 75 cm. All clay fraction grain size measurements were performed using a LS2000 Beckman Coulter granulometer.*

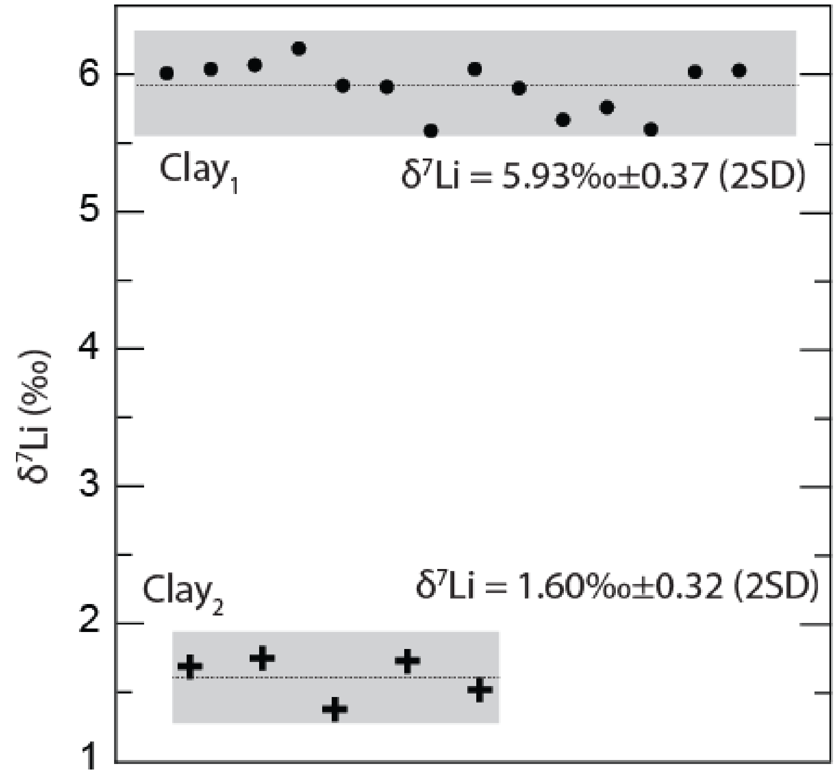

Figure S2: *Repeatability of Li isotope measurements performed for two clay samples*.

Table S1: *δ^7Li values measured on various standard solutions and clay particles.*

	Without chemical purification	With chemical purification				
	Li^7N	Li^6N	Li^7N	Seawater	Clay1	Clay2
	30.27	-7.65	30.46	31.08	6.03	1.72
	30.22	-7.77	30.09	31.22	6.06	1.37
	30.35	-8.05	30.18	31.10	6.09	1.74
	29.84		30.40	30.77	6.21	1.68
	30.01		30.30	31.04	5.94	1.51
	30.43		30.28		5.93	
	30.24		30.16		5.61	
	30.31		29.88		6.06	
	29.97		30.48		5.92	
	30.29		30.39		5.69	
	29.93		30.24		5.78	
	30.18		30.36		5.62	
	30.42		30.41		6.04	
	29.93				6.05	
	30.04					
	30.48					
	30.34					
	30.36					
Mean	30.2	-7.82	30.2	31.0	5.9	1.6
2σn	0.1	0.2	0.1	0.1	0.4	0.3

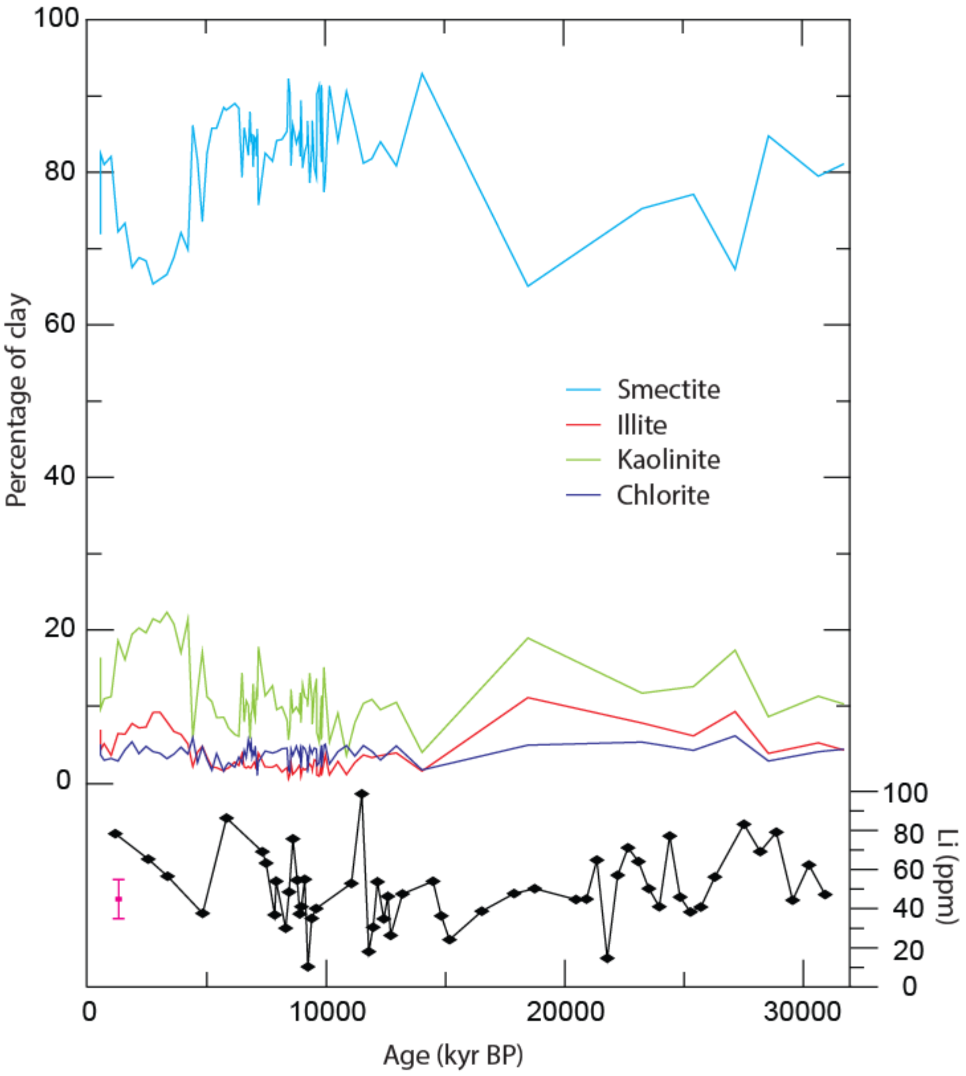

Figure S3: *Mineralogical composition of the clay-sized fraction estimated by XRD as a function of time*[14] *and the lithium concentration in the clay fraction (black, this study). The pink bar corresponds to the mean value and uncertainties of lithium concentrations determined for clay samples collected in rivers of the Ethiopian traps, and submitted to the same physical and chemical protocol (Bastian et al., in prep).*

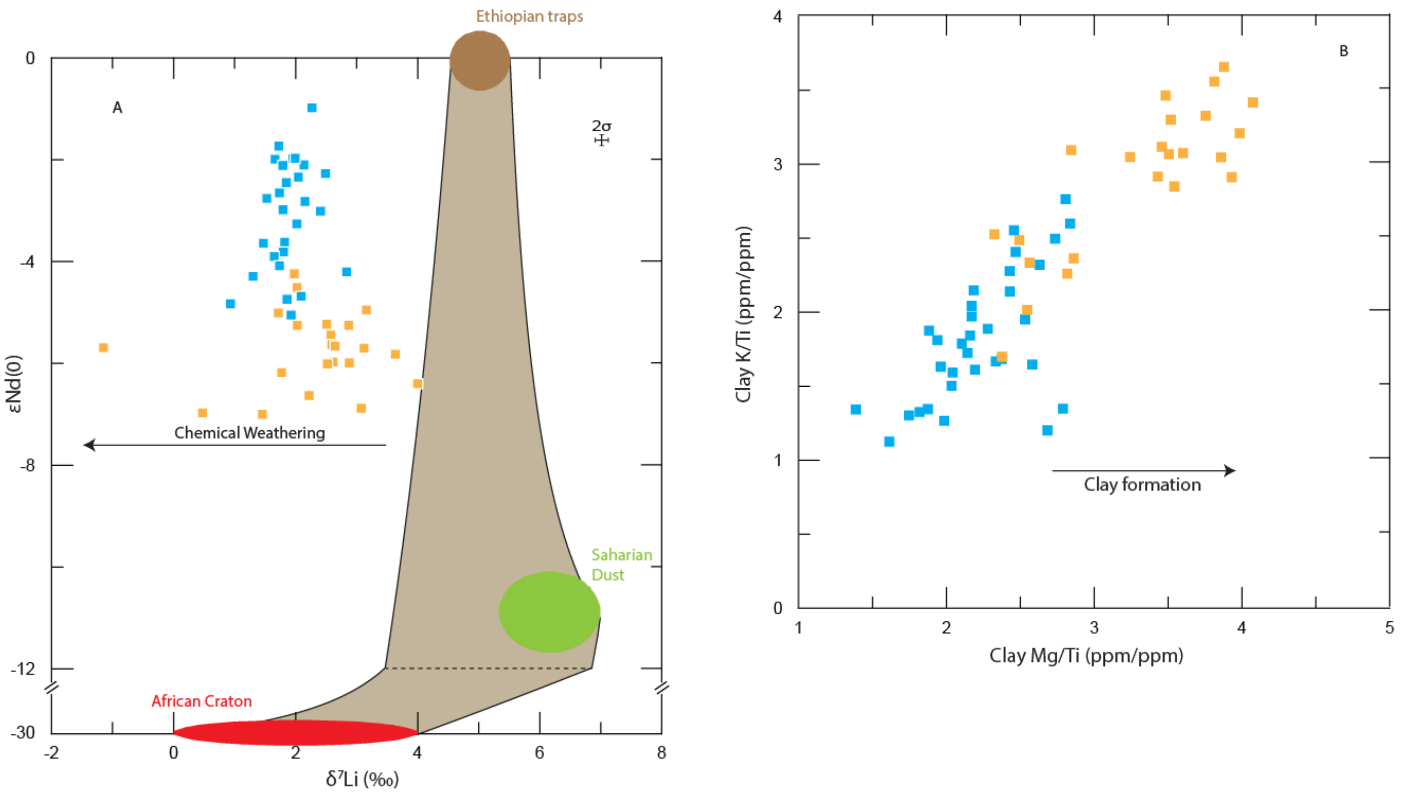

Figure S4: *(A) Clay lithium isotope composition as a function of clay neodymium isotope composition (MS27PT). The brown zone corresponds to the mixing zone for source rocks from the Ethiopian Traps, the African craton and the Saharan Dust (based on measured values (Table S1) and published average values for each endmember). The Saharan dust samples have been collected in the Libyan desert by Francis Grousset and consists mostly in quartz and carbonate grains (pers. comm.)*[13]*. The average d7Li value is 6.2±0.8‰ and average Li content is 2.2 ppm +/- 0.5ppm. The Ethiopian traps samples have been collected by Raphael Pik. Their average d7Li value is 5.0±0.5‰ with a Li content of 13.9ppm +/- 0.8 ppm. Clay* δ^7*Li are systematically enriched in* ^{6}*Li compared to fresh source rocks due to isotope fractionation during the clay formation process; (B) Clay K/Ti ratios as a function of clay Mg/Ti (MS27PT). The blue squares and orange squares correspond to humid and arid periods, respectively.*

Modeling Li isotopes

All published studies indicate significant lithium isotopic fractionation during secondary phase formation, in particular clay formation. This isotopic fractionation is always in favor of the light isotope (^{6}Li) and does not depend on the clay type[27]. In contrast, rock or mineral leaching is not associated with significant isotope fractionation at low temperature. Several recent studies have modeled the behavior of Li isotopes at the scale of a watershed or of a continent according to the following equations[27–31]:

$$F^{Li}_{lea}\delta_{lea} = F^{Li}_{sp}\delta_{sp} + F^{Li}_{riv}\delta_{riv} \qquad \text{equation (1)}$$

$$\delta_{\mathrm{sp}} = \delta_{\mathrm{riv}} - \Delta_{\mathrm{land}} \qquad \text{equation (2)}$$

$$\delta_{lea} = \delta_{\mathrm{BR}} \qquad \text{equation (3)}$$

$$\delta_{\mathrm{sp}} = \frac{\Delta_{\mathrm{land}}}{\mathrm{F}_{lea}^{\mathrm{Li}} / \mathrm{F}_{\mathrm{sp}}^{\mathrm{Li}}} + \delta_{\mathrm{BR}} - \Delta_{\mathrm{land}} \qquad \text{equation (4)}$$

where $\mathrm{F}_{lea}^{\mathrm{Li}}$, $\mathrm{F}_{\mathrm{sp}}^{\mathrm{Li}}$, and $\mathrm{F}_{\mathrm{riv}}^{\mathrm{Li}}$ are the flux of lithium during dissolution, in the secondary phase and in the river water, respectively, and δ_{lea}, δ_{sp}, δ_{riv} and δ_{BR} are the Li isotopic signature of the dissolved phase, the secondary phase, the river water and the bedrock, respectively. This model corresponds to a mass balance for lithium, such that, the Li released by the dissolution/leaching of the source rocks ($\mathrm{F}_{lea}^{\mathrm{Li}}$) is either incorporated into secondary minerals during their neoformation or released into the rivers, as shown in the box model in Figure S5. $\mathrm{F}_{lea}^{\mathrm{Li}}$ and $\mathrm{F}_{\mathrm{sp}}^{\mathrm{Li}}$ depend on Li concentrations of the bedrock and the clays respectively, as well as dissolution rates and clay neoformation rates. Bedrock leaching is assumed to be isotopically congruent while clay neoformation fractionate Li isotopes (Δ_{land})[29,30]. This model is similar as the one developed by[29] which describes a conceptual model of alteration in the weathering zone and apply it to several isotope systems (e.g. Li, Mg, Ca, Si). The mass balance written for Li in equation (1) implies that the dissolved Li inventory and isotope composition are at steady state, which is likely to be true beyond a certain timescale related to the water residence time in the system. Equation (1) does not necessarily imply a soil "steady-state", as defined by several authors[29,32] as a balance between physical denudation and soil production by chemical erosion (constant soil thickness). Indeed, if soil thickness is increasing, due to low physical erosion and more neoformation, then more Li is immobilized into the weathering profile (in clays or secondary phases), and less Li is released into the river and if soil thickness is decreasing, more Li is released into rivers. Theoretical modeling curves are reported in Figure S5 for various clay-water isotope fractionation factors. As shown in this graph, during humid periods, the clay δ^7Li values (δ_{sp}) measured in the MS27PT core are in general low and homogeneous. This is consistent with the modelling and is best explained by a high $\mathrm{F}_{lea}^{\mathrm{Li}} / \mathrm{F}_{\mathrm{sp}}^{\mathrm{Li}}$ ratio and a low clay-

water isotope fractionation factor. During arid periods, the clay δ^7Li values (δ_{sp}) are much more variable, increasing to higher values at specific times such as during the LGM. According to the model, this corresponds to lower F_{lea}^{Li}/F_{sp}^{Li} ratios. When the F_{lea}^{Li}/F_{sp}^{Li} ratio is low, the slope of the curve is steeper, and therefore clay δ^7Li values are more sensitive to small variations in the alteration regime (F_{lea}/F_{sp}). In contrast, during humid periods (blue rectangles), fluctuations in the alteration regime do not affect significantly the clay δ^7Li values. This is consistent with the differences in the variability of our data between humid and arid periods (see text for more details).

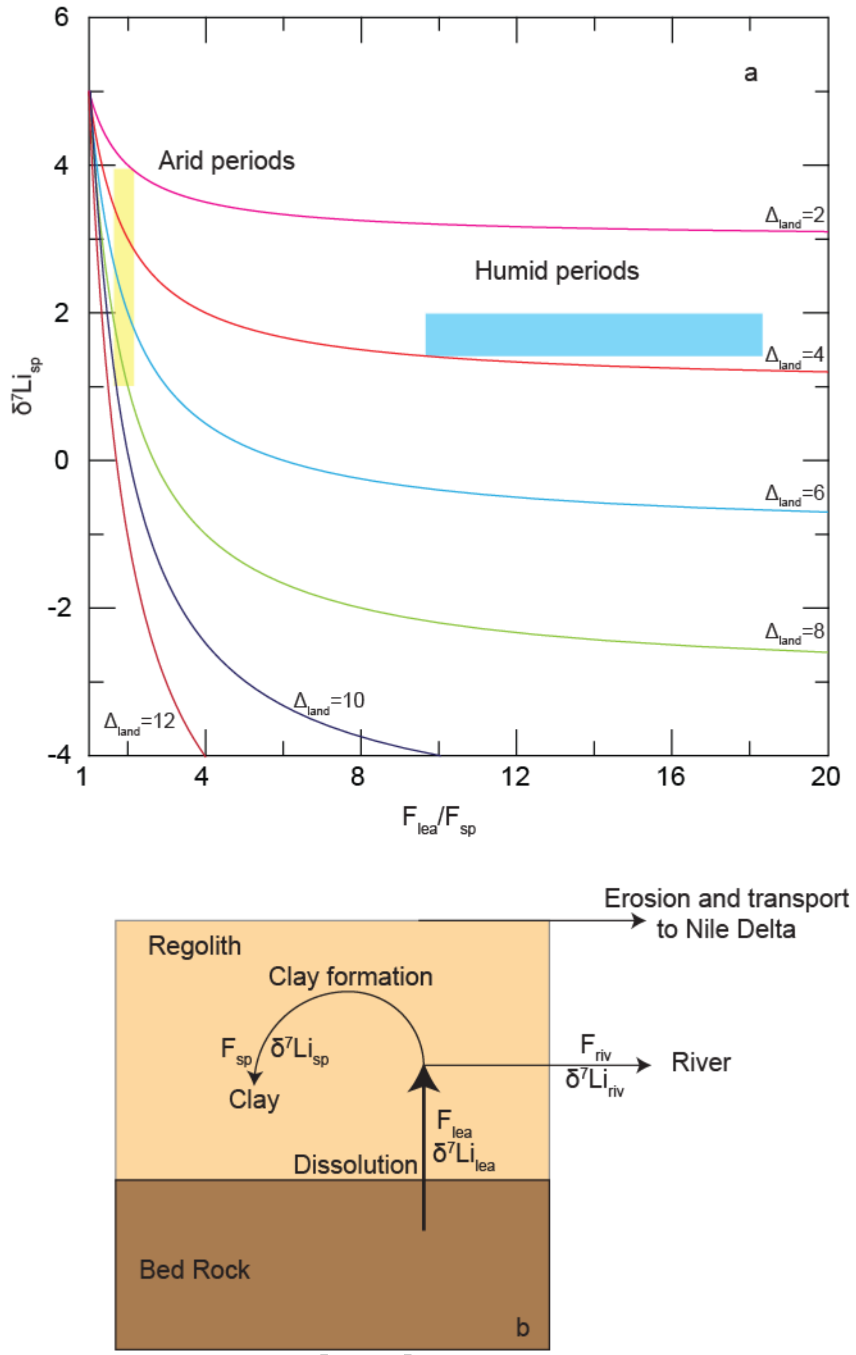

Figure S5: *a) Theoretical curves of δ^7Li_{sp} (δ^7Li value of secondary phases) as a function of F_{lea}/F_{sp}, where F_{lea} and F_{sp} are the Li flux corresponding to the dissolution of the source rock, and the flux of Li incorporated into secondary phase, respectively. Δ_{land} is the clay-water isotope fractionation. The yellow and blue shaded fields represent the range of clay δ^7Li values determined for the arid and humid periods, respectively. Their x-axis positions have been adjusted arbitrarily in order to illustrate the fact that, for a single isotope fractionation factor, a small range of clay δ7Li value, as exhibited during humid periods, is expected at high F_{lea}^{Li}/F_{sp}^{Li} values, while a larger range correspond to lower ratios. It also illustrates why during humid periods, large variations of F_{lea}^{Li}/F_{sp}^{Li}, ratio does not impact significantly the clay δ7Li value. In contrast, during arid periods, small variations of dissolution / precipitation rates result in significant clay δ7Li variations. b) Conceptual view of lithium fluxes taken into account in the modelling.*

***Table S2**: Li and Nd isotopic data and major/trace element data measured in the clay fractions of core MS27PT. Depth corresponds to the distance from the top of the core. The age was determined by interpolation between two calculated ^{14}C ages (Table S2 from [10]), under the assumption that the sedimentary rate has not varied. The errors for K, Ca, Mg, Fe, Mn, Al, Ti, Sr, Ba and Li are 3.5% 3.4%, 3.4%, 1.6%, 5.3%, 2.3%, 2.8%, 2.9 % and 4.3% respectively.*

Depth (cm)	Age (years)	δ7Li (‰)	2σr	εNd	2σr	K (ppm)	Ca (ppm)	Mg (ppm)	Fe (ppm)	Mn (ppm)	Al (ppm)	Ti (ppm)	Sr (ppm)	Ba (ppm)	Li (ppm)
2	1258	-1.15	0.01	-5.69	0.10	10435	6825	13161	58063	468	83496	5178	99	118	78
7	2640	0.47	0.06	-6.97	0.14	9462	5593	11442	48771	406	74363	4004	86	107	65
11	3449	1.45	0.01	-7.00	0.20	10085	7900	12547	49196	650	75360	4460	84	90	57
17	4923	1.72	0.01	-5.01	0.12	8345	7419	11648	55481	1345	80416	4911	74	81	38
22	5925	2.02	0.06	-4.51	0.16	8809	6125	13807	62372	2837	86411	5353	117	252	86
34.5	7430	1.47	0.02	-3.64	0.13	8389	7569	10136	48903	230	76140	4449	102	265	69
39	7582	1.85	0.02	-2.45	0.14	6962	2312	9987	56083	261	83422	6196	43	94	63
51.5	7946	1.82	0.02	-3.62	0.17	6415	8341	9053	38324	220	62691	3814	84	594	37
53.5	8007	1.80	0.01	-3.81	0.12	8011	1855	10854	54539	250	87305	5338	41	175	54
70	8403	2.11	0.06			2699	683	2789	20011	74	25328	2013	22	72	30
82	8551	2.14	0.01	-2.10	0.13	7276	2140	10143	51683	241	79639	5413	43	91	49
95	8712	2.47	0.04			10940	2639	13154	65099	267	100518	6710	90	187	76
102	8891	2.83	0.02	-4.20	0.19	4637	2170	9598	32073	207	48923	3444	46	104	55
113.5	8999	2.49	0.01	-2.27	0.12	7825	2754	10646	49649	256	77660	4858	44	116	37
120	9068	1.88	0.08			10607	2927	13182	63673	299	95435	6158	68	151	41
136.5	9203	1.79	0.01	-2.98	0.17	6509	2582	8734	46204	234	75645	5001	61	114	55
152	9338	2.41	0.02	-3.01	0.23	2729	998	6099	21531	114	33565	2274	23	52	10
171.5	9498	1.72	0.01	-1.73	0.17	6640	2765	10416	44665	199	73400	5251	60	108	35
193.5	9683	2.04	0.01	-2.34	0.27	7820	1721	10735	59852	257	86246	5908	45	101	40
206	9820	2.43	0.06			11950	3456	13159	68776	307	91734	6068	2	2	
243	11165	1.81	0.05			10816	2839	12732	69625	312	91808	6054	87	159	53
255	11601	1.89	0.06			13414	2741	13641	74538	397	92617	6248	127	273	99
263	11892	2.27	0.02	-0.98	0.17	6953	2843	9738	48456	339	60775	4174	51	88	18
268.5	12074	2.15	0.01	-2.82	0.20	12553	11335	13754	54371	410	76112	5031	93	127	30
273.5	12256	1.73	0.01	-2.65	0.23	12537	4342	14230	65449	322	90769	5862	76	131	54
278	12522	1.66	0.02	-1.99	0.12	10692	5139	12533	61840	316	83253	5806	72	121	35
281	12695	1.53	0.02	-2.76	0.11	13116	5430	13444	65636	378	85482	5451	92	162	46
283	12810	1.79	0.02	-2.11	0.15	10838	4141	14068	70606	382	80655	5559	62	99	26
288.5	13309	1.99	0.02	-1.97	0.20	11872	3253	12705	67458	286	92166	6559	89	157	48
295.5	14588	1.62	0.02	-3.65	0.14	13727	4547	13765	59401	281	85995	5524	99	156	54
296	14934	1.98	0.04	-4.24	0.12	13352	8779	14661	57803	337	83986	5726	100	123	36
298	15280	2.58	0.02	-5.44	0.17	12504	3860	11508	48161	210	75342	4954	74	115	24
299.5	16635	2.03	0.02	-5.25	0.16	13222	6947	14097	44666	272	71637	4008	108	144	39
300	17989	3.08	0.02	-6.88	0.14	16766	6817	16858	56043	294	89104	4842	99	137	48
301	18855	2.59	0.01	-5.63	0.22	14297	5036	13137	51575	274	82681	4623	93	144	50
303	20588	2.61	0.02	-5.97	0.15	15128	16233	16098	53555	429	83988	4966	145	149	45
304	21024	2.22	0.03	-6.63	0.31	19571	7884	20968	69762	379	106095	5502	109	156	45
305	21461	2.52	0.04	-6.01	0.14	19954	7058	21161	71853	388	107552	5459	123	177	65
306	21897	3.63	0.01	-5.82	0.16	5985	4543	8080	25019	184	37725	2056	60	55	15
307	22334	2.88	0.04	-5.99	0.14	18428	8853	21972	72584	430	104919	5396	121	166	57
308	22770	4.00	0.03	-6.40	0.23	18886	6742	21309	73860	385	112993	5679	85	133	71
309.5	23207	2.65	0.01	-5.66	0.18	14487	17469	16560	50266	416	80330	4727	95	101	64
310	23643	2.51	0.03	-5.23	0.12	15381	22738	18010	54098	662	80417	5004	138	167	50
311	24079	2.87	0.03	-5.25	0.12	13407	23702	15773	54185	649	79622	4600	163	134	41
312	24516	3.16	0.04	-4.95	0.08	15861	26783	19724	60148	945	88750	5571	173	203	77
313	24952	3.12	0.04	-5.70	0.14	14771	42122	18343	51323	1033	78292	4605	81	124	46
314	25389	1.77	0.08	-6.18	0.16	16059	8943	20345	64657	742	93881	5275	197	128	38
315.5	25825	0.93	0.01	-4.83	0.18	15025	13890	16667	59873	481	84287	4823	94	105	41
317	26411	1.92	0.01	-5.05	0.16	13742	4293	13213	62907	305	91155	5386	67	120	56
321	27630	1.65	0.04	-3.90	0.16	14988	4251	15207	80426	366	96004	5426	93	167	83
323	28312	1.86	0.04	-4.74	0.27	15271	4348	16659	79788	376	104102	5880	75	143	69
325	28994	1.74	0.01	-4.08	0.33	8514	2887	9038	48445	265	66676	4172	98	180	79
327	29677	2.09	0.03	-4.68	0.45	13396	10921	15181	78665	396	98034	5779	89	127	44
329	30359	2.02	0.02	-3.26	0.12	14354	4242	15286	83886	364	108094	6307	80	151	62
331	31041	1.30	0.01	-4.29	0.18	11904	3095	11938	65601	303	93432	6354	66	134	47

Table S3: *Radiocarbon chronology of core MS27PT*[10]*. Radiocarbon dating was performed at the Laboratoire de Mesure du Carbone 14-Saclay (Paris, France) by the national facility in the framework of INSU ARTEMIS call-for-proposal.*

Depth (cm)	Lab code	Species	**Corrected age (yr BP)**	$^{14}C_{error}$	Age cal. 1s	Age cal. 1s	**Cal Bp age median probability**
0.5	SacA005001	G. ruber alba	**1060**	**35**	564	589	**577**
2.5	SacA31699	G. ruber alba	**1695**	**30**	1238	1278	**1258**
5.5	SacA22673	G. ruber alba	**2670**	**30**	2306	2344	**2325**
10.5	SacA22674	G. ruber alba	**3345**	**30**	3064	3161	**3113**
13.5	SacA16511	G. ruber alba	**4160**	**30**	4084	4157	**4121**
22	SacA16513	G. ruber alba	**5570**	**30**	5908	5941	**5925**
23.5	SacA22675	G. ruber alba	**5795**	**30**	6235	6274	**6255**
25	SacA16514	G. ruber alba	**6055**	**30**	6406	6466	**6436**
29.5	SacA005003	G. ruber alba	**6415**	**50**	6791	6913	**6852**
34.5	SacA31700	G. ruber alba	**6900**	**30**	7415	7444	**7430**
65	SacA10935	G. ruber alba	**7945**	**30**	8352	8389	**8371**
70	SacA11797	G. ruber alba	**8010**	**30**	8388	8417	**8403**
95.5	SacA11798	G. ruber alba	**8330**	**30**	8644	8779	**8712**
102	SacA11799	G. ruber alba	**8385**	**30**	8861	8921	**8891**
120	SacA11800	G. ruber alba	**8520**	**30**	9049	9086	**9068**
205.5	SacA10936	G. ruber alba	**9185**	**35**	9735	9832	**9784**
274	SacA16516	G. ruber alba	**10835**	**40**	12234	12349	**12292**
284	SacA16517	G. ruber alba	**11495**	**40**	12913	13050	**12982**
293.5	SacA10937	G. ruber alba	**12795**	**45**	14056	14198	**14127**
298	SacA31702	Bulk forams?	**13630**	**60**	15172	15388	**15280**
300.5	SacA31703	Bulk forams?	**15590**	**60**	17898	18080	**17989**
303	SacA16518	G. ruber alba	**17470**	**70**	20484	20691	**20588**
315	SacA10938	G. ruber alba	**21920**	**80**	25742	25907	**25825**
333	SacA11801	G. ruber alba	**27760**	**130**	31128	31329	**31229**

Supplementary Material References

1. Ghilardi, M. & Boraik, M. Reconstructing the holocene depositional environments in the western part of Ancient Karnak temples complex (Egypt): a geoarchaeological approach. *J. Archaeol. Sci.* **38,** 3204–3216 (2011).

2. Garzanti, E., Andò, S., Padoan, M., Vezzoli, G. & El Kammar, A. The modern Nile sediment system: Processes and products. *Quat. Sci. Rev.* **130,** 9–56 (2015).

3. Williams, M. A. J. *et al.* Late Quaternary environments and prehistoric occupation in the lower White Nile valley, central Sudan. *Quat. Sci. Rev.* **130,** 72–88 (2015).

4. Verschuren, D. *et al.* Half-precessional dynamics of monsoon rainfall near the East African Equator. *Nature* **462,** 637–41 (2009).

5. Costa, K., Russell, J., Konecky, B. & Lamb, H. Isotopic reconstruction of the African Humid Period and Congo Air Boundary migration at Lake Tana, Ethiopia. *Quat. Sci. Rev.* **83,** 58–67 (2014).

6. Shanahan, T. M. *et al.* The time-transgressive termination of the African Humid Period. *Nat. Geosci.* **2,** 1–5 (2015).

7. Tuenter, E., Weber, S. L., Hilgen, F. J. & Lourens, L. J. The response of the African summer monsoon to remote and local forcing due to precession and obliquity. *Glob. Planet. Change* **36,** 219–235 (2003).

8. DeMenocal, P. & Tierney, J. E. Green Sahara: African Humid Periods Paced by Earth's Orbital Changes. *Nat. Educ. Knowl.* **3,** 12 (2012).

9. deMenocal, P. *et al.* Abrupt onset and termination of the African Humid Period : rapid climate responses to gradual insolation forcing. *Quat. Sci. Rev.* **19,** 347–361 (2000).

10. Revel, M. *et al.* 20,000 years of Nile River dynamics and environmental changes in the Nile catchment area as inferred from Nile upper continental slope sediments. *Quat. Sci. Rev.* **130,** 200–221 (2015).

11. Williams, M. *et al.* Abrupt return of the summer monsoon 15,000 years ago: new supporting evidence from the lower White Nile valley and Lake Albert. *Quat. Sci. Rev.* **25,** 2651–2665 (2006).

12. Ducassou, E. *et al.* Nile floods recorded in deep Mediterranean sediments. *Quat. Res.* **70,** 382–391 (2008).

13. Revel, M. *et al.* 100,000 Years of African monsoon variability recorded in sediments of the Nile margin. *Quat. Sci. Rev.* **29,** 1342–1362 (2010).

14. Revel, M. *et al.* 21,000 Years of Ethiopian African monsoon variability recorded in sediments of the western Nile deep-sea fan. *Reg. Environ. Chang.* **14,** 1685–1696 (2014).

15. Migeon, S. *et al.* Lobe construction and sand/mud segregation by turbidity currents and debris flows on the western Nile deep-sea fan (Eastern Mediterranean). *Sediment. Geol.* **229,** 124–143 (2010).

16. Blanchet, C. L. *et al.* High- and low-latitude forcing of the Nile River regime during the Holocene inferred from laminated sediments of the Nile deep-sea fan. *Earth Planet. Sci. Lett.* **364,** 98–110 (2013).

17. Deschamps, P. *et al.* Ice-sheet collapse and sea-level rise at the Bølling warming 14,600 years ago. *Nature* **483,** 559–564 (2012).

18. Marriner, N. *et al.* ITCZ and ENSO-like pacing of Nile delta hydro-geomorphology during the Holocene. *Quat. Sci. Rev.* **45,** 73–84 (2012).

19. Reimer, P. J. *et al.* Intcal13 and Marine13 Radiocarbon Age Calibration Curves 0–50,000 Years Cal Bp. *Radiocarbon* **55,** 1869–1887 (2013).

20. Siani, G. *et al.* Mediterranean Sea surface radiocarbon reservoir age changes since the last glacial maximum. *Science (80-.).* **294,** 1917–1920 (2001).

21. Labeyrie, L., Sinko, J. A., Bond, G., Duprat, J. & Cortijo, E. Patterns of IceRafted Detritus in the Glacial North Atlantic (40-55°N). *Paleoceanography* **8,** 175–192 (1993).

22. Bond, G. *et al.* Correlations between climate record from North Atlantic sediments and Greenland ice. *Nature* **365,** 143–147 (1993).

23. Martrat, B., Jimenez-Amat, P., Zahn, R. & Grimalt, J. O. Similarities and dissimilarities between the last two deglaciations and interglaciations in the North Atlantic region. *Quat. Sci. Rev.* **99,** 122–134 (2014).

24. Bahr, A. *et al.* Persistent monsoonal forcing of mediterranean outflow water dynamics during the late Pleistocene. *Geology* **43,** 951–954 (2015).

25. Collins, J. A. *et al.* Abrupt shifts of the Sahara-Sahel boundary during Heinrich stadials. *Clim. Past* **9,** 1181–1191 (2013).

26. Naughton, F. *et al.* Wet to dry climatic trend in north-western Iberia within Heinrich events. *Earth Planet. Sci. Lett.* **284,** 329–342 (2009).

27. Dellinger, M. *et al.* Riverine Li isotope fractionation in the Amazon River basin controlled by the weathering regimes. *Geochim. Cosmochim. Acta* **164,** 71–93 (2015).

28. Vigier, N., Gislason, S. R., Burton, K. W., Millot, R. & Mokadem, F. The relationship between riverine lithium isotope composition and silicate weathering rates in Iceland. *Earth Planet. Sci. Lett.* **287,** 434–441 (2009).

29. Bouchez, J., Von Blanckenburg, F. & Schuessler, J. A. Modeling novel stable isotope ratios in the weathering zone. *Am. J. Sci.* **313,** 267–308 (2013).

30. Vigier, N. & Goddéris, Y. A new approach for modeling Cenozoic oceanic lithium isotope paleo-variations: The key role of climate. *Clim. Past* **11,** 635–645 (2015).

31. Li, G. & West, a. J. Evolution of Cenozoic seawater lithium isotopes: Coupling of global denudation regime and shifting seawater sinks. *Earth Planet. Sci. Lett.* **401,** 284–293 (2014).

32. Gaillardet, J., Dupre, B., Louvat, P. & Allegre, C. J. Global silicate weathering and CO 2 consumption rates deduced from the chemistry of large rivers. *Chem. Geol.* **159,** 3–30 (1999).
